# Bacterial contamination in the different parts of household air conditioners: a comprehensive evaluation from Chengdu, Southwest China

**DOI:** 10.3389/fpubh.2024.1429626

**Published:** 2024-08-14

**Authors:** Donglei Sun, Li Tang, Keyan Long, Weilian Sun, Zunzhen Zhang

**Affiliations:** West China School of Public Health and West China Fourth Hospital, Sichuan University, Chengdu, China

**Keywords:** microbial contamination, household air-conditioner, indoor environment, bacterium, 16S ribosomal RNA gene sequencing

## Abstract

**Introduction:**

Air flow driven by air-conditioner has a significant impact on the indoor environment, however, the bacterial contamination conditions in the different parts of air-conditioners have not been fully elucidated.

**Methods:**

In this study, we assessed the bacterial pollution in the four parts, including air outlet, filter net, cooling fin and water sink, of ten household air-conditioners quantitatively and qualitatively from Chengdu, southwestern China.

**Results:**

The microbial cultivation results showed the large total bacterial counts of 5042.0, 9127.6, 6595.1, and 12296.2 CFU/cm^2^ in air outlet, filter net, cooling fin, and water sink. Furthermore, the sequencing data showed that these four parts displayed different bacterial characteristics. At the level of genus, *Caproiciproducens* and *Acidipropionibacterium* were predominant in air outlet. *Bacillus, Acinetobacter, Paracoccus*, and *Corynebacterium* were detected as the characteristic bacteria in filter net. For cooling fin, *Rhodococcus, Achromobacter*, and *Nocardioides* were the dominant bacteria. The genera of *Methylobacterium-Methylorubrum, Brevibacterium, Stenotrophomonas*, and *Psychrobacter* were identified as the bioindicators in water sink. The bioinformatic analysis on the sequencing data illustrated that the bacteria from air-conditioners were associated with metabolic disturbance.

**Discussion:**

This study reveals the distinct bacterial compositions in the different parts of air-conditioner, and provides new clues for the non-negligible bacterial pollution in this common appliance from Chinese households.

## 1 Introduction

With worsening global warming, the demand for air conditioners has soared in recent years. Air conditioner sales in China rose from 51.5 million units in 2010 to nearly 100 million units in 2020 (1), and China has been the world's largest country of air conditioner consumption (2). It is generally accepted that people spend almost 90% of their time in indoor environments (3), and a long use duration of air conditioners has been reported in Chinese households (4). The internal moist environment in air conditioners provides ideal living conditions for the growth and survival of various bacteria (5). Compared with only natural-ventilated buildings, sick building syndrome (SBS) morbidity in buildings with air conditioners has increased by 30–200% (6). It has been reported that there is a significant association between using an air conditioner and the presence and severity of asthma and rhinitis (7). The outbreak of Legionnaires' disease (221 cases with 34 deaths) in Philadelphia in 1976 aroused people's attention to the microbial contamination of air conditioners (8). Moreover, the frequent detection of Severe Acute Respiratory Syndrome Coronavirus 2 (SARS-CoV-2) in air-conditioning filters in recent years has highlighted the microbial contamination in air-conditioning systems (9).

Few studies to date have focused on the bacterial pollution of air conditioners in residential households. The multitudinous bacteria released by air conditioners have been detected and reported in several developed countries, including the USA (10), Japan (11), and Singapore (12). Abundant bacteria, including pathogens, could be loaded on air conditioner filter microfibers, which widely exist in indoor air and would pose health risks (13). More seriously, the pathogenic phylum of *Proteobacteria* is present in greater proportion in air conditioner filter dust samples than in other dust samples from indoor environments (14). These findings all suggested that the air conditioner was a non-negligible source of indoor bacterial contamination. Compared with developed countries, Chinese residents generally lack awareness of cleaning household appliances, which may promote the breeding and spread of microorganisms in air conditioners.

Currently, the sampling part in the daily monitoring of air conditioner hygiene by the Centers for Disease Control and Prevention is air outlets (15). Apart from air outlets, air conditioners also have internal structures, such as air filters and cooling and water sinks. Each part of the air conditioner has different temperature and relative humidity conditions (16), which may result in distinct bacterial features. The cooling coil of the air conditioner has been found to become the source and colonization of bacteria (5, 17, 18). A recent study from Shanghai revealed bacterial contamination in residential air-conditioning filters (19). Interestingly, distinct characteristic bacteria have been found in the cooling coil and air filter (20), suggesting bacterial diversity in the different parts. Distinguishing the bacterial bioindicators in the different parts using high-throughput sequencing would provide a comprehensive understanding of the bacterial contamination of air conditioners.

In this study, we hypothesized that air conditioners were prone to microbial contamination and that different parts had distinct bacterial compositions. We performed a combination of microbial cultivation and 16S ribosomal ribonucleic acid (RNA) gene sequencing to explore the bacterial pollution in the air outlet, filter net, cooling fin, and water sink of air conditioners in the megacity of Chengdu, Southwest China. This study uncovers the detailed bacterial diversity in these parts and provides a basic clue for air conditioners as a bacterial contamination source in indoor environments in Chinese households.

## 2 Materials and methods

### 2.1 Study design and sample collection

The study site was set in Chengdu, Southwest China. Samples were collected from 10 household air conditioners, including five cabinet air conditioners and five wall-hanging air conditioners, from September to November 2022 using convenience sampling. Each air conditioner had four sampling parts: an air outlet, a filter net, a cooling fin, and a water sink. The information about these air conditioners was recorded through a user questionnaire. Microbial samples were collected using a 5 × 5 cm (25 cm^2^) sterile specification board with S-shaped smearing by a cotton swab soaked with 1 mL saline. The smeared part of the cotton swab was cut off into the sample tube with 9 mL 0.9% saline solution, and the dilatability of the original sample was regarded as 10^−1^. We collected samples before and after a thorough air conditioner cleaning through the disassembly of inner parts by a professional commercial company, respectively.

### 2.2 Detection of total bacterial count

The total bacterial count was detected for the four parts of the air conditioner using the plate count method according to Chinese national standard GB/T 18204.4-2013 (21) to reflect the sanitary conditions. Briefly, the original smeared samples (regarding 10^−1^ dilatability) were diluted into 10^−2^, 10^−3^, 10^−4^, and 10^−5^. Subsequently, 1 mL of each diluted sample was added to a plate with two parallels, followed by 20 mL 45°C nutrient agar medium (Luqiao Biological Technology Co. Ltd., Beijing, China) poured onto the plate. The plates were then incubated at 37°C for 48 h. Colony counts of 30 and 300 colony-forming units (CFUs) in plates were counted, and the bacterial count unit for each sample was calculated using the following equation based on the two adjacent dilutions (Equation 1):


(1) $$\begin{array}{l} N=\frac{\sum C}{\left(n_{1}+0.1n_{2}\right)d}/S \end{array}$$

*N* is the colony-forming unit, CFU/cm^2^, ∑*C* is the sum of the colonies on the plates, *n*_1_ is the plate number of low dilatability and the value is 2, *n*_2_ is the plate number of high dilatability and the value is 2, *d* is the dilution factor of low dilatability, and *S* is the sampling area of 25 cm^2^.

If the plates in a single dilution had a colony count of 30–300, the colony-forming unit was directly calculated by the Equation (2):


(2) $$N=\frac{\left(C_{1}+C_{2}\right)}{2}\times b/S$$

Where *N* is the colony-forming unit, CFU/cm^2^, *C*_1_, and *C*_2_ are the number of colonies on the parallel plates, *b* is the fold of dilution, and *S* is the sampling area of 25 cm^2^.

### 2.3 Measurement of total fungal count

A total fungal count in the air conditioner was also evaluated using the plate count method according to the Chinese national standard GB/T 18204.4-2013 (21) to reflect the sanitary conditions. The original samples were regarded as having 10^−1^ dilatability and the samples were further diluted into 10^−2^, 10^−3^, and 10^−4^. Then, 1 mL of each diluted sample was added to a plate with two parallels, followed by 20 mL of 45°C Rose Bengal Medium (Luqiao Biological Technology Co. Ltd., Beijing, China) poured onto the plate. Afterwards, the plates were incubated at 27°C for 1 week. Colony counts between 5 and 50 colony-forming units (CFUs) in plates within the same dilution were counted, and the fungi count unit was calculated by the Equation (3):


(3) $$N=\frac{\left(C_{1}+C_{2}\right)}{2}\times b/S$$

Where *N* is the colony-forming unit, CFU/cm^2^, *C*_1_, and *C*_2_ are the number of colonies on the parallel plates, *b* is the fold of dilution, and *S* is the sampling area of 25 cm^2^.

### 2.4 DNA extraction and 16S ribosomal RNA (rRNA) gene sequencing

A total of 40 samples of 4 parts from the 10 air conditioners before the machine cleaning were filtered by a 0.22 μm sterile filter membrane and the total microbial DNA extraction was performed using a commercial kit (Tiangen Biotech, Beijing Co., Ltd., China) according to the manufacturer's instructions. DNA concentration and purity were determined using a NanoDrop 2000 spectrophotometer (Thermo Fisher Scientific, Rochester, NY, USA). All samples were sent to Majorbio Biotech (Shanghai, China) for 16S rRNA gene sequencing. The sequencing data were deposited in the National Center for Biotechnology Information (NCBI) Sequence Read Archive database and the BioProject accession number was PRJNA1028381.

### 2.5 Microbial diversity analysis

The sequencing data were analyzed by the Majorbio online system (https://cloud.majorbio.com/). The coverage index was used to reflect the real situation of the microorganisms detected in the samples. Alpha-diversity reflected bacterial diversity and richness and was expressed as the Chao index and Shannon index in this study by Mothur (version 1.30.2). Beta diversity showed the differences in bacterial composition between samples or groups. We used principal coordinate analysis (PCoA), principal component analysis (PCA), and non-metric multidimensional scaling (NMDS) to show beta diversity in this study. In addition, analysis of similarities (ANOSIM) and partial least squares discriminant analysis (PLS-DA) were used to compare microbial diversity between the different groups.

### 2.6 Cluster analysis

A Venn diagram was used to show the number of microbial intersections between the four groups at the genus level. We used the barplot of the bacterial community to express the relative bacterial abundance of the 40 samples, or the four groups, at the phylum level. A Linear discriminant analysis Effect Size (LEfSe) barplot was used to identify specific species that were differentially distributed between the four groups at the genus level. For LEfSe analysis, the linear discriminant analysis (LDA) score threshold was set at >3.0. A circular phylogenetic tree at the genus level was used to show the taxonomic bacterial profiling of the 40 samples. The detailed evolutionary relationship and expression differences of the microbial community between the four groups were also shown in a phylogenetic tree at the genus level.

### 2.7 Prediction of phenotype and gene function

Metabolic pathway analysis by Clusters of Orthologous Groups (COGs) of proteins and functional annotation by the Kyoto Encyclopedia of Genes and Genomes (KEGG) were performed to analyze the enriched pathways of bacterial genes from the air conditioners. These data suggest the potential effects of bacterial contamination from air conditioners on human health. We also used BugBase to annotate microbiome phenotypes, including stress tolerance, mobile elements, Gram-negative, Gram-positive, and biofilms.

### 2.8 Statistical analysis

The results were illustrated as the average value ± standard deviation. The difference in microbial cultivation before and after the air conditioner cleaning was analyzed using a paired sample *t*-test. Differences in microbial culture results, alpha diversity and microbial phenotype of the four sampling parts were analyzed using one-way analysis of variance (ANOVA), followed by the Student–Newman–Keuls *post-hoc* test. Beta diversity was expressed based on weighted UniFrac matrices. We performed the ANOVA analysis using the Statistical Package for the Social Sciences (SPSS; IBM Inc., Chicago, IL, USA), version 21.0. A statistical significance was set at *P* < 0.05. The sequencing data were statistically analyzed by the Majorbio online system.

## 3 Results

### 3.1 Data information on collected samples

**Figure 1:**
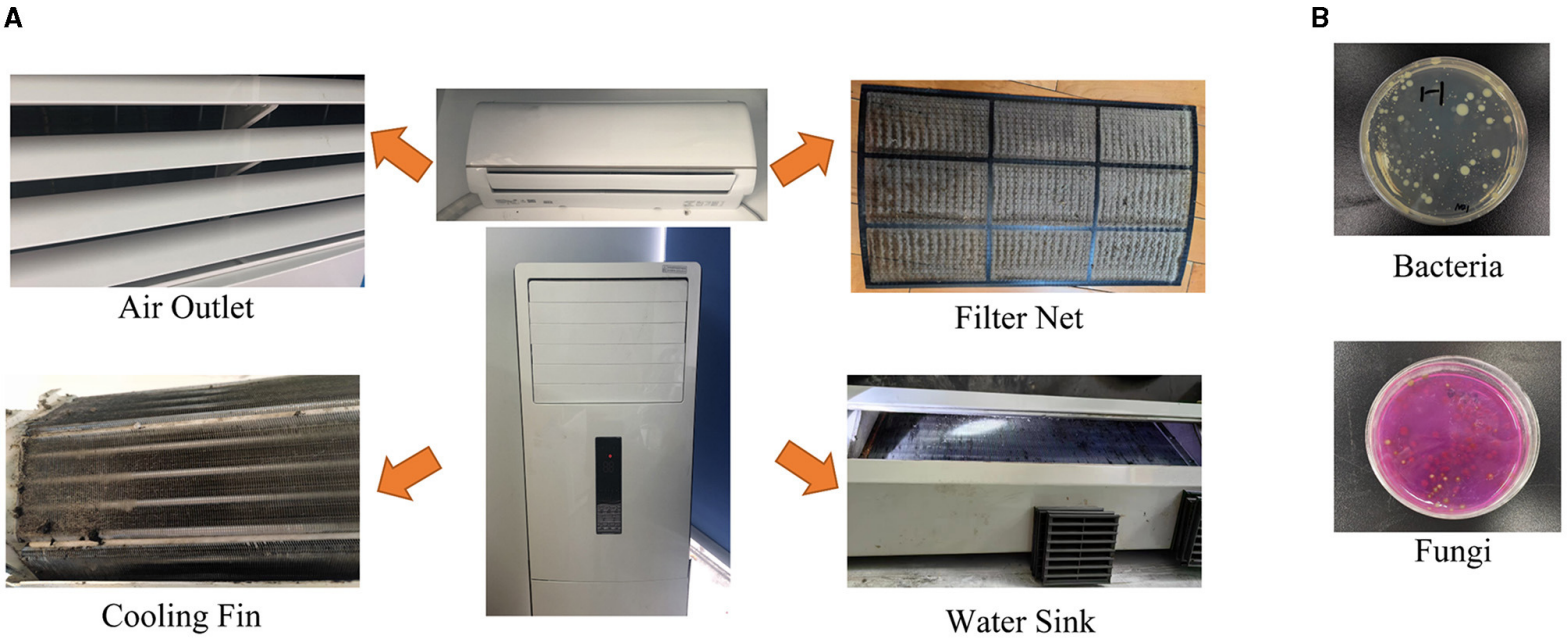
The sampling parts of air conditioners and the representative images of microbial cultivation. **(A)** The samples from the air outlet, filter net, cooling fin, and water sink were collected with soaked cotton swabs. **(B)** Representative result images of bacteria and fungi.

In our study, 40 samples were collected from the air outlet, filter net, cooling fin, and water sink of 10 air conditioners from 10 households in Chengdu, Southwest China (Figure 1A). The samples were collected before and after a thorough machine cleaning by the disassembly of inner parts, respectively (Table 1). There were five cabinet air conditioners and five wall-hanging air conditioners in this study (Table 2). The information on sample ID, using years and the last machine cleaning time was also listed in Table 2.

**Table 1:** Information on the number of samples and the detection indexes.

**Sampling parts**	**The number of samples**		**Testing indexes**	
	**Before cleaning** ^*^	**After cleaning**	**Before cleaning**	**After cleaning**
Air outlet	10	10	Total bacterial count, total fungi count, 16S rRNA gene sequencing	Total bacterial count, total fungi count
Filter net	10	10		
Cooling fin	10	10		
Water sink	10	10		

^*^A thorough machine cleaning by the disassembly of inner parts.

**Table 2:** Sample ID and the data of using years.

**Household**	**Mode**	**Using years**	The last machine cleaning time^*^	**Sample ID**	
				**Prior to the machine cleaning**	**After the machine cleaning**
1	Cabinet	>1	0–3 years	AC01A	AC01B
2	Cabinet	>3	>3 years	AC02A	AC02B
3	Cabinet	>2	0–3 years	AC03A	AC03B
4	Cabinet	>6	>3 years	AC04A	AC04B
5	Cabinet	>5	>3 years	AC05A	AC05B
6	Wall-hanging	>3	0–3 years	AC06A	AC06B
7	Wall-hanging	>3	0–3 years	AC07A	AC07B
8	Wall-hanging	>2	0–3 years	AC08A	AC08B
9	Wall-hanging	>4	>3 years	AC09A	AC09B
10	Wall-hanging	>5	>3 years	AC10A	AC10B

^*^A thorough machine cleaning by the disassembly of inner parts.

### 3.2 Microbial counts in the different parts of air conditioners

Representative images of microbial cultivation are shown in Figure 1B. The average total bacterial counts were 5,042.0 ± 1,149.8, 9,127.6 ± 2,053.5, 6,595.1 ± 1,253.8, and 12,296.2 ± 711.7 CFU/cm^2^ in the air outlet, filter net, cooling fin, and water sink before the air conditioner cleaning, respectively (Table 3 and Supplementary Table 1). For total fungal count, the average numbers were 818.0 ± 305.1, 1,462.0 ± 269.4, 1,080.0 ± 258.7, and 1,650.0 ± 231.9 CFU/cm^2^ in the air outlet, filter net, cooling fin, and water sink before the machine cleaning, respectively (Table 3 and Supplementary Table 2). As expected, the machine cleaning of the air conditioner by disassembly of the inner parts remarkably reduced bacterial and fungal counts in all samples (Table 3), indicating the importance of daily machine cleaning in reducing microbial contamination of domestic air conditioners.

**Table 3:** The data of microbial culture results.

**Sampling parts**	**Total bacterial count (CFU/cm** ^**2**^ **)**		**Total fungi count (CFU/cm** ^**2**^ **)**	
	**Before cleaning^a^**	**After cleaning**	**Before cleaning**	**After cleaning**
Air outlet	5,042.0 ± 1,149.8^***^	15.3 ± 2.4	818.0 ± 305.1^***^	7.7 ± 3.2
Filter net	9,127.6 ± 2,053.5^***^	15.6 ± 1.7	1,462.0 ± 269.4^***^	10.0 ± 2.0
Cooling fin	6,595.1 ± 1,253.8^***^	15.7 ± 1.3	1,080.0 ± 258.7^***^	10.5 ± 3.3
Water sink	12,296.2 ± 711.7^***^	19.2 ± 2.7	1,650.0 ± 231.9^***^	11.5 ± 1.8

Data are presented the means ± SD.

^a^A thorough machine cleaning by the disassembly of inner parts.

^***^Compared with the count after the air-conditioner machine cleaning, P < 0.001.

**Figure 2:**
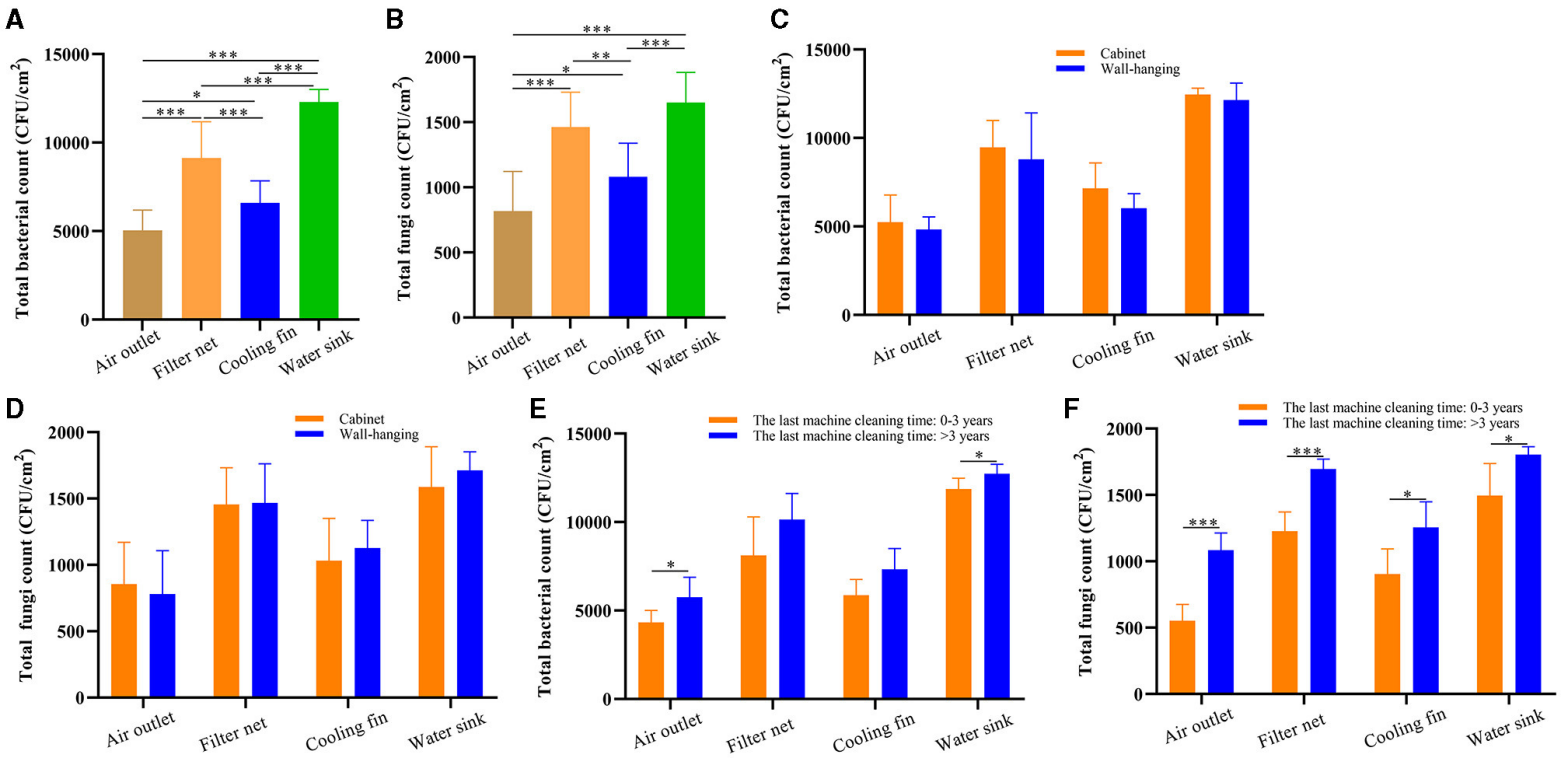
The microbial culture results of different sampling parts in air conditioners. **(A)** Total bacterial count in the air outlet, filter net, cooling fin, and water sink. **(B)** Total fungal count in the air outlet, filter net, cooling fin, and water sink. **(C)** Total bacterial count in the different parts of the air conditioners: cabinet and wall-hanging. **(D)** Total fungal count in the different parts of the air conditioners: cabinet and wall-hanging. **(E)** Total bacterial count of the last machine cleaning time >3 and 0–3 years in the different sampling parts. **(F)** Total fungal count of the last machine cleaning time >3 and 0–3 years in the different sampling parts. *P < 0.05, **P < 0.01, ***P < 0.001, compared to the corresponding group indicated by the horizontal line.

We further analyzed the difference in microbial count between the four sampling parts, including the air outlet, filter net, cooling fin, and water sink. The total bacterial count was the highest in the water sink, followed by the filter net, cooling fin, and air outlet (Figure 2A). Moreover, the water sink had the highest total fungal count, and the air outlet had the lowest total fungal count (Figure 2B). In addition, total bacterial and fungal counts showed no difference between the cabinet air conditioners and wall-hanging air conditioners in all sampling parts (Figures 2C, D). Furthermore, the total bacterial count was significantly increased in the group of the last machine cleaning time >3 years compared to the group of 0–3 years in the air outlet and water sink (Figure 2E). For total fungal count, the numbers were significantly increased in the group of the last machine cleaning time >3 years compared to the group of 0–3 years in the four sampling parts (Figure 2F). Taken together, total bacterial and fungal counts were expressed differently in the different parts of the air conditioner and increased with the increase in uncleaned years.

### 3.3 Different parts of the air conditioner had distinct microbial diversity

**Figure 3:**
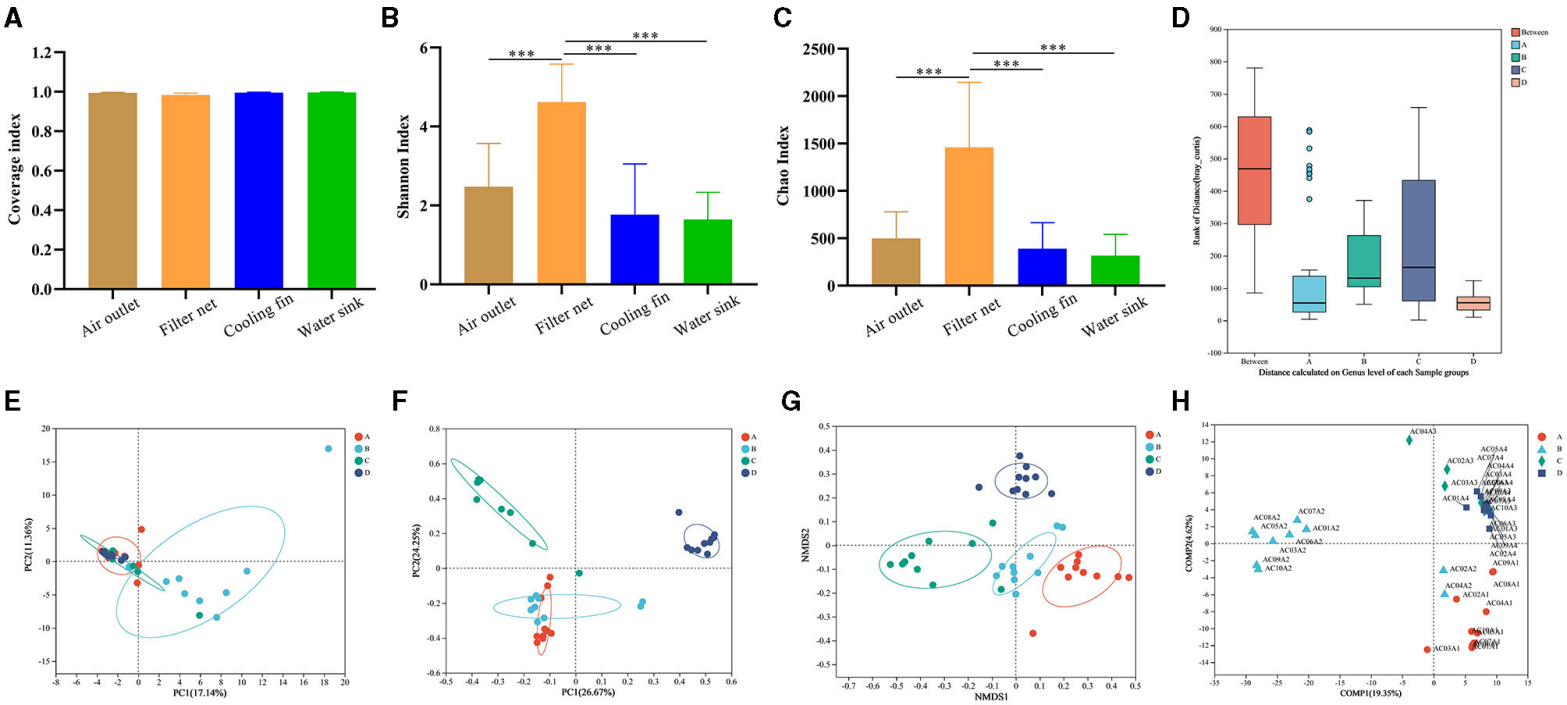
Comparisons of the bacterial diversity in the different parts of air conditioners. **(A)** Coverage index to show alpha diversity. **(B)** Shannon index to show alpha diversity. **(C)** Chao index to show alpha diversity. **(D)** Analysis of similarities (ANOSIM). **(E)** Principal component analysis (PCA) to show beta diversity. **(F)** Principal coordinate analysis (PCoA) to show beta diversity. **(G)** Non-metric multidimensional scaling (NMDS) to show beta diversity. **(H)** Partial least squares discriminant analysis (PLS-DA). Group A: air outlet; Group B: filter net; Group C: cooling fin; and Group D: water sink. Sample ID: AC (air conditioner) + The household number + A (before the machine cleaning) + Sampling parts (1 represented air outlet, 2 represented filter net, 3 represented cooling fin, 4 represented water sink). ***P < 0.001, compared to the corresponding group indicated by the horizontal line.

The bacterial composition of 50 samples before the machine cleaning was further detected to provide insight into the microbial contamination of the air conditioner by using 16S rRNA gene sequencing. The sample ID in the gene sequencing is listed in Supplementary Table 3. The raw data were deposited in the NCBI Sequence Read Archive database, and the BioProject accession number was PRJNA1028381 (https://www.ncbi.nlm.nih.gov/bioproject/?term=PRJNA1028381). The values of the coverage index were higher than 99.0% in all groups of the four sampling parts (Figure 3A), indicating the high quality of the sequencing data. As indicators of alpha diversity, the Chao index and Shannon index reflected species richness. In this study, the values of the Chao index and Shannon index in the filter net were significantly higher (*P* < 0.001) than the groups of air outlet, cooling fin, and water sink (Figures 3B, C).

At the genus level, analysis of similarities (ANOSIM) showed that the difference between the four parts was significantly greater (*P* < 0.01) than the difference within each group (Figure 3D), indicating that bacteria in these parts had their own composition characteristics. We further used diverse beta-diversity indexes to explore the difference in community compositions between the different groups in the four sampling parts. PCA results showed that the samples of filter net had a different bacterial composition from the other three groups (Figure 3E). In PCoA analysis, samples of the cooling fin and water sink showed distinct bacterial compositions, while there was an overlap between the air outlet and filter net (Figure 3F). In addition, NMDS results showed that each group had a separate circle of community composition (Figure 3G). The PLS-DA results displayed the detailed distribution of bacterial composition for each sample (Figure 3H). Taken together, these results indicated that different parts of the air conditioner had distinct microbial diversity.

### 3.4 Different parts of the air conditioner had characteristic microbial bioindicators

**Figure 4:**
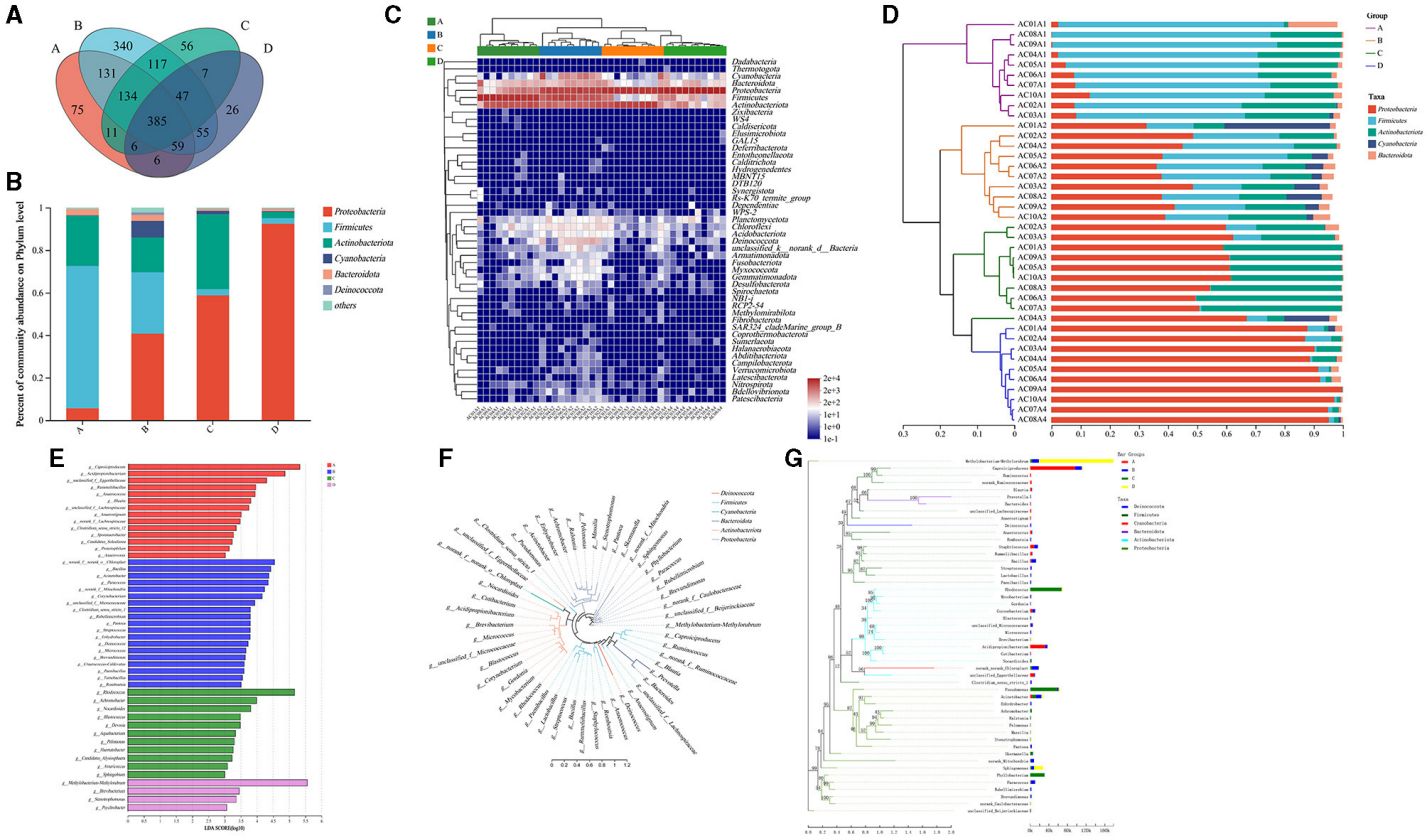
Analysis of the microbial composition among different parts of air conditioners. **(A)** The Venn diagram at the phylum level. **(B)** Community barplot analysis of groups at the phylum level. **(C)** Community heatmap at the phylum level. **(D)** Community barplot analysis of samples at the phylum level. **(E)** Linear discriminant analysis Effect Size (LEfSe) barplot at various taxonomic levels. **(F)** Circular phylogenetic tree at the genus level. **(G)** Phylogenetic tree analysis based on the sequencing reads of different groups. Group A: air outlet; Group B: filter net; Group C: cooling fin; Group D: water sink. f-, o-, c-, and p- represented the level of family, order, class, and phylum, respectively.

Overall, 48 phyla, 133 classes, 324 orders, 571 families, 1,455 genera, and 2,918 species were detected in this study. The four sampling parts showed an overlap of 385 genera (Figure 4A). The group of water sinks showed the highest relative abundance of *Proteobacteria* (Figure 4B), followed by the cooling fin, filter net, and air outlet. In addition, the phyla of *Firmicutes, Actinobacteriota*, and *Cyanobacteria* had different relative abundances in each group (Figure 4B). The community heatmap at the phylum level showed detailed information on relative abundance for each sample and illustrated that *Proteobacteria, Actinobacteriota, Bacteroidota, Firmicutes*, and *Cyanobacteria* were dominant in the air conditioner (Figure 4C). Notably, the hierarchical clustering tree further showed that *Bacteroidota* and *Cyanobacteria* had a relatively high abundance in filter net samples (Figure 4D).

The bacterial indicators for each group at the genus level were further analyzed. LEfSe barplot showed that the genus *Caproiciproducens* (belonging to the family *Ruminococcaceae*, phylum *Firmicutes*) and the genus *Acidipropionibacterium* (belonging to the family *Propionibacteriaceae*, phylum *Actinobacteriota*) were the predominant bacteria in the group of air outlets (Figure 4E). The genus *Bacillus* (belonging to the family *Bacillaceae*, phylum *Firmicutes*), the genus *Acinetobacter* (belonging to the family *Moraxellaceae*, phylum *Proteobacteria*), the genus *Paracoccus* (belonging to the family *Rhodobacteraceae*, phylum *Proteobacteria*), and the genus *Corynebacterium* (belonging to the family *Corynebacteriaceae*, phylum *Actinobacteriota*) had relatively high abundance in the filter net (Figure 4E).

For cooling fins, the genus *Rhodococcus* (belonging to the family *Nocardiaceae*, phylum *Actinobacteriota*), the genus *Achromobacter* (belonging to the family *Alcaligenaceae*, phylum *Proteobacteria*), and the genus *Nocardioides* (belonging to the family *Nocardioidaceae*, phylum *Actinobacteriota*) were the dominant bacteria (Figure 4E). Furthermore, the genus *Methylobacterium-Methylorubrum* (belonging to the family *Beijerinckiaceae*, phylum *Proteobacteria*), the genus *Brevibacterium* (belonging to the family *Brevibacteriaceae*, phylum *Actinobacteriota*), the genus *Stenotrophomonas* (belonging to the family *Xanthomonadaceae*, phylum *Proteobacteria*) and the genus *Psychrobacter* (belonging to the family *Moraxellaceae*, phylum *Proteobacteria*) expressed predominantly in water sinks (Figure 4E). The circular phylogenetic tree showed the evolutionary difference between the dominant bacteria at the genus level (Figure 4F). At the genus level, the combination of evolutionary relationships and expression differences of important bacteria between the four groups was shown in a phylogenetic tree graph (Figure 4G). All these results illustrated that different parts of the air conditioner had distinct bacterial bioindicators.

### 3.5 Potential adverse effects of bacteria from air conditioners on residents

**Figure 5:**
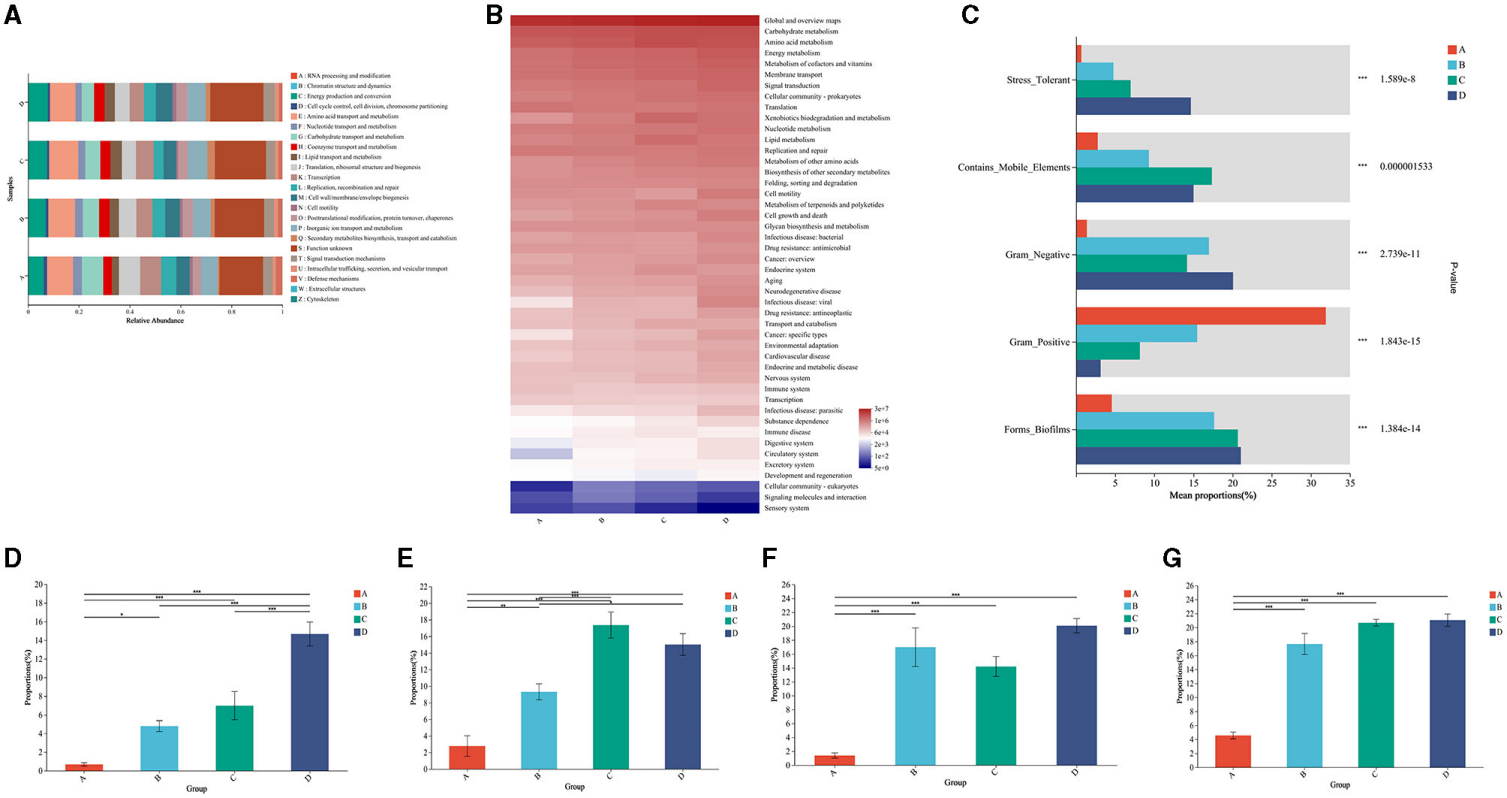
Functional profiling of the microbial community in air conditioners. **(A)** Analysis of metabolic pathways by Clusters of Orthologous Groups (COG) of proteins. **(B)** Functional annotation by the Kyoto Encyclopedia of Genes and Genomes (KEGG). **(C)** Functional profiling between the different parts. **(D)** The comparison of stress tolerance between the different groups. **(E)** The comparison of mobile elements between the different groups. **(F)** The comparison of Gram-negative bacteria between the different groups. **(G)** The comparison of biofilm formation between the different groups. Group A: air outlet; Group B: filter net; Group C: cooling fin; Group D: water sink. *P < 0.05, **P < 0.01, ***P < 0.001, compared to the corresponding group indicated by the horizontal line.

The potential adverse effects of bacteria from air conditioners on residents were further analyzed by the bioinformatic method. The COG function classification showed that the bacteria from the air conditioner were associated with amino acid transport and metabolism, carbohydrate transport and metabolism, lipid transport and metabolism, and energy production and conversion (Figure 5A). Accordingly, KEGG analysis illustrated that the bacteria from the air conditioner were tightly associated with the pathways of carbohydrate metabolism, amino acid metabolism, energy metabolism, metabolism of cofactors and vitamins, bacterial infectious disease, and antimicrobial drug resistance (Figure 5B).

For microbial phenotypes, bacteria from the different parts of the air conditioner had distinct characteristics of being stress-tolerant, mobile elements, Gram-negative, Gram-positive, and biofilms (Figure 5C). Specifically, bacteria showed an increased trend in stress tolerance in the air outlet, filter net, cooling fin, and water sink (Figure 5D). Bacteria from the cooling fin and water sink showed a higher ability to move than the parts of the air outlet and filter net (Figure 5E). Gram-negative bacteria had the least proportion in the air outlet (Figure 5F). Moreover, bacteria from the filter net, cooling fin, and water sink showed a high ability to form biofilms (Figure 5G). All these results suggested the potential adverse effects of bacteria from air conditioners on residents.

## 4 Discussion

**Figure 6:**
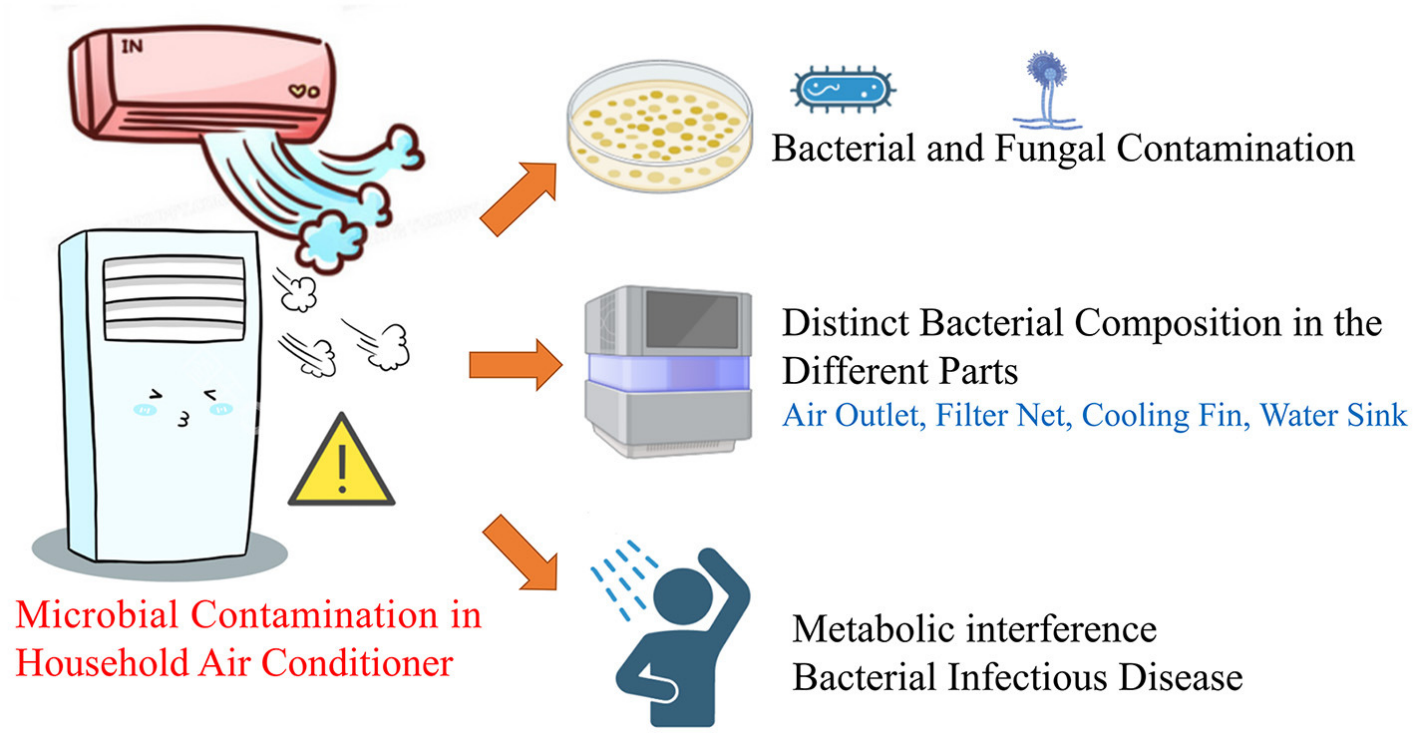
Microbial pollution in the different parts of Chinese household air-conditioner.

Currently, microorganisms released by air conditioners in hospital wards are strictly monitored and controlled (22, 23). Moreover, the hygiene of air conditioners in public places is monitored by the Centers for Disease Control and Prevention. However, in household settings, evaluations of the bacterial pollution of air conditioners have only been reported in a few studies from developed countries (10–12). At present, the data on microbial pollution in Chinese air conditioners is limited. Our study highlighted the microbial contamination in household air conditioners and further identified the distinct bacterial features in the air outlet, filter net, cooling fin, and water sink, advancing novel insights on the air conditioner as a microbe intermediary for the indoor environment in Chinese households (Figure 6).

Total bacterial and fungal counts reflect the overall microbiological hygiene and are strictly restricted to < 500 CFU/cm^2^ on the surface of public air-conditioning outlets in the Chinese hygienic standard of GB/T 18204.5-2013 (24). However, the microbial count in the household environment has not been regulated. In this study, the total bacterial and fungal counts from household air conditioners greatly exceeded the limits regulated by hygienic standard GB/T 18204.5-2013. The average total bacterial counts were 5,042.0, 9,127.6, 6,595.1, and 12,296.2 CFU/cm^2^ in the air outlet, filter net, cooling fin, and water sink before the air conditioner was cleaned, respectively (Table 3 and Supplementary Table 1). These results were consistent with the bacterial concentration of 10^3^-10^4^ copies/cm^2^ reported by the study from Shanghai (19), indicating the severe microbial pollution of Chinese household air conditioners.

As the most common sampling part, the average bacterial DNA concentrations on the filter surface of the air conditioner were reported to be 0.02–3.3 ng per cm^2^ from a Singaporean study (12). US research reported that the median bacterial concentration from air conditioner filter dust was 1.1 × 10^6^ CFU/g (14). Our study further suggested that samples from the filter net showed a higher bacterial richness than the other three parts of the air outlet, cooling fin, and water sink. For the species composition, our results were consistent with published sequencing analysis that *Proteobacteria, Actinobacteria*, and *Firmicutes* were the common phylum in air conditioners (11, 12, 20). Our PCoA results were similar to those of a Japanese study where air outlet and filter net samples were plotted close together (20). In this study, decentralized air conditioners were sampled, and *Legionella* was not detected in our sequencing data. Indeed, it is often suggested that *Legionella* is frequently detected in public centralized air-conditioning systems rather than in household decentralized air conditioners (25). Moreover, *Legionella* might have been outcompeted by other predominant bacteria in our samples. In addition, a handful of non-tuberculous *Mycobacteria* (NTM) species have been implicated in pulmonary diseases (26) and can be transmitted through the central air-conditioning systems in public buildings (27). Although NTM species were not detected in our study, the risk of strain distribution in household air conditioners cannot be neglected.

*Caproiciproducens* and *Acidipropionibacterium* were found predominantly in air outlets and filter nets in our study. These genera are associated with malodor formation (28, 29) and have been detected in washing machines in our recently published study (30). *Bacillus, Acinetobacter, Paracoccus*, and *Corynebacterium* had relatively high abundances in the filter net. *Bacillus* and *Acinetobacter* strains have been found to cause localized wound and eye infections (31, 32). Moreover, *Paracoccus* and *Corynebacterium* have been reported to colonize skin lesions (33–35). For the biomarkers found in cooling fins, *Rhodococcus* and *Achromobacter* have been recognized as pathogenic bacteria (36, 37). For the bioindicators found in the water sink, *Methylobacterium-Methylorubrum* colonizes moist areas and can survive in harsh environments (38). It has been reported that *Brevibacterium, Stenotrophomonas*, and *Psychrobacter* serve as global opportunistic pathogens (39–41).

Increasing evidence has shown that the energy metabolism of carbohydrates, amino acids, and lipids plays a vital role in immunometabolic crosstalk during bacterial infection (42, 43). The bacterial interference on metabolism in indoor environments has raised growing concerns in recent years (44–46). Our results suggested the potential risk of bacterial disturbance in human metabolism from the source of the air conditioner. Bacteria with the structure of biofilm can be protected and acquire resistance against harsh environmental conditions (47). In addition, airborne antibiotic resistance has raised growing concerns in indoor environments (48). The results of the biofilm phenotype further suggested that the air conditioner served as a bacterial source for indoor microbial contamination.

Admittedly, there were two limitations in this study. First, the causal relationship between pathogenic bacteria identified in this study and indoor infection has not been demonstrated and needs further study. Second, several factors, such as climate conditions and usage habits, were not considered in this study.

## 5 Conclusion

This study revealed the severe bacterial and fungal pollution of household air conditioners in Chengdu, Southwest China, suggesting this appliance is a non-ignorable microbial contamination source. The characteristic bacterial genera of air outlet, filter net, cooling fin, and water sink were further identified, representing a fundamental understanding of the distinct bacterial compositions at the different sites of air conditioners. This study reminds the public of the importance of the hygiene of household air conditioners.

## Data availability statement

The datasets presented in this study can be found in online repositories. The names of the repository/repositories and accession number(s) can be found in the article/Supplementary material.

## Author contributions

DS: Conceptualization, Funding acquisition, Methodology, Writing – original draft. LT: Methodology, Writing – review & editing. KL: Investigation, Methodology, Writing – review & editing. WS: Software, Writing – review & editing. ZZ: Supervision, Writing – review & editing.
