# Supplementary material for: Bacterial contamination in the different parts of household air conditioners: a comprehensive evaluation from Chengdu, Southwest China
Source: Front Public Health. 2024 Aug 14;12:1429626. doi: 10.3389/fpubh.2024.1429626 (PMC11350112; doi:10.3389/fpubh.2024.1429626)
Supplement: Supplementary file 1 [file Table_1.docx]

S1. The data of total bacterial count for all samples.

| Sample ID | Sampling part | Dilutability | | | | | Colony forming unit  (CFU/cm^2^, round to single digits^)^ |
| --- | --- | --- | --- | --- | --- | --- | --- |
|  |  | 10^-1^ | 10^-2^ | 10^-3^ | 10^-4^ | 10^-5^ |  |
| AC01A | Air outlet | >300, >300^a^ | >300, >300 | 110, 96 | <30, <30 | 0, 0 | 4120 |
|  | Filter net | >300, >300^a^ | >300, >300 | 236, 248 | 48, 55 | 0, 0 | 10672 |
|  | Cooling fin | >300, >300^a^ | >300, >300 | 181, 175 | <30, <30 | 0, 0 | 7120 |
|  | Water sink | >300, >300^a^ | >300, >300 | 268, 280 | 67, 52 | 0, 0 | 12127 |
| AC01B | Air outlet | 36, 31 | 0, 0 | 0, 0 | 0, 0 | 0, 0 | 13 |
|  | Filter net | 33, 35 | 0, 0 | 0, 0 | 0, 0 | 0, 0 | 14 |
|  | Cooling fin | 37, 36 | 0, 0 | 0, 0 | 0, 0 | 0, 0 | 15 |
|  | Water sink | 42, 38 | 0, 0 | 0, 0 | 0, 0 | 0, 0 | 16 |
| AC02A | Air outlet | >300, >300^a^ | >300, >300 | 186, 200 | <30, <30 | 0, 0 | 7720 |
|  | Filter net | >300, >300^a^ | >300, >300 | 245, 233 | 37, 40 | 0, 0 | 10091 |
|  | Cooling fin | >300, >300^a^ | >300, >300 | 208, 212 | 33, 36 | 0, 0 | 8891 |
|  | Water sink | >300, >300^a^ | >300, >300 | 278, 290 | 76, 72 | 0, 0 | 13018 |
| AC02B | Air outlet | 32, 30 | 0, 0 | 0, 0 | 0, 0 | 0, 0 | 12 |
|  | Filter net | 37, 44 | 0, 0 | 0, 0 | 0, 0 | 0, 0 | 16 |
|  | Cooling fin | 35, 38 | 0, 0 | 0, 0 | 0, 0 | 0, 0 | 15 |
|  | Water sink | 40, 46 | 0, 0 | 0, 0 | 0, 0 | 0, 0 | 17 |
| AC03A | Air outlet | >300, >300^a^ | >300, >300 | 90, 104 | <30, <30 | 0, 0 | 3880 |
|  | Filter net | >300, >300^a^ | >300, >300 | 197, 205 | <30, <30 | 0, 0 | 8040 |
|  | Cooling fin | >300, >300^a^ | >300, >300 | 136, 138 | <30, <30 | 0, 0 | 5480 |
|  | Water sink | >300, >300^a^ | >300, >300 | 287, 294 | 53, 58 | 0, 0 | 12582 |
| AC03B | Air outlet | 31, 33 | 0, 0 | 0, 0 | 0, 0 | 0, 0 | 13 |
|  | Filter net | 36, 32 | 0, 0 | 0, 0 | 0, 0 | 0, 0 | 14 |
|  | Cooling fin | 38, 34 | 0, 0 | 0, 0 | 0, 0 | 0, 0 | 14 |
|  | Water sink | 56, 43 | 0, 0 | 0, 0 | 0, 0 | 0, 0 | 20 |
| AC04A | Air outlet | >300, >300^a^ | >300, >300 | 115, 132 | <30, <30 | 0, 0 | 4940 |
|  | Filter net | >300, >300^a^ | >300, >300 | 188, 193 | <30, <30 | 0, 0 | 7620 |
|  | Cooling fin | >300, >300^a^ | >300, >300 | 149, 155 | <30, <30 | 0, 0 | 6080 |
|  | Water sink | >300, >300^a^ | >300, >300 | 276, 279 | 65, 48 | 0, 0 | 12145 |
| AC04B | Air outlet | 42, 41 | 0, 0 | 0, 0 | 0, 0 | 0, 0 | 17 |
|  | Filter net | 35, 36 | 0, 0 | 0, 0 | 0, 0 | 0, 0 | 14 |
|  | Cooling fin | 42, 45 | 0, 0 | 0, 0 | 0, 0 | 0, 0 | 17 |
|  | Water sink | 42, 47 | 0, 0 | 0, 0 | 0, 0 | 0, 0 | 18 |
| AC05A | Air outlet | >300, >300^a^ | >300, >300 | 142, 137 | <30, <30 | 0, 0 | 5580 |
|  | Filter net | >300, >300^a^ | >300, >300 | 247, 266 | 46, 40 | 0, 0 | 10891 |
|  | Cooling fin | >300, >300^a^ | >300, >300 | 194, 218 | <30, <30 | 0, 0 | 8240 |
|  | Water sink | >300, >300^a^ | >300, >300 | 263, 282 | 75, 62 | 0, 0 | 12400 |
| AC05B | Air outlet | 35, 42 | 0, 0 | 0, 0 | 0, 0 | 0, 0 | 15 |
|  | Filter net | 44, 49 | 0, 0 | 0, 0 | 0, 0 | 0, 0 | 19 |
|  | Cooling fin | 37, 48 | 0, 0 | 0, 0 | 0, 0 | 0, 0 | 17 |
|  | Water sink | 56, 52 | 0, 0 | 0, 0 | 0, 0 | 0, 0 | 22 |

Table continued.

| Sample ID | Sampling part | Dilutability | | | | | Colony forming unit  (CFU/cm^2^, round to single digits^)^ |
| --- | --- | --- | --- | --- | --- | --- | --- |
|  |  | 10^-1^ | 10^-2^ | 10^-3^ | 10^-4^ | 10^-5^ |  |
| AC06A | Air outlet | >300, >300^a^ | >300, >300 | 95, 112 | <30, <30 | 0, 0 | 4140 |
|  | Filter net | >300, >300^a^ | >300, >300 | 143, 138 | <30, <30 | 0, 0 | 5620 |
|  | Cooling fin | >300, >300^a^ | >300, >300 | 112, 130 | <30, <30 | 0, 0 | 4840 |
|  | Water sink | >300, >300^a^ | >300, >300 | 254, 258 | 46, 49 | 0, 0 | 11036 |
| AC06B | Air outlet | 40, 34 | 0, 0 | 0, 0 | 0, 0 | 0, 0 | 15 |
|  | Filter net | 36, 38 | 0, 0 | 0, 0 | 0, 0 | 0, 0 | 15 |
|  | Cooling fin | 41, 37 | 0, 0 | 0, 0 | 0, 0 | 0, 0 | 16 |
|  | Water sink | 48, 44 | 0, 0 | 0, 0 | 0, 0 | 0, 0 | 18 |
| AC07A | Air outlet | >300, >300^a^ | >300, >300 | 135, 141 | <30, <30 | 0, 0 | 5520 |
|  | Filter net | >300, >300^a^ | >300, >300 | 226, 235 | 45, 37 | 0, 0 | 9873 |
|  | Cooling fin | >300, >300^a^ | >300, >300 | 145, 174 | <30, <30 | 0, 0 | 6380 |
|  | Water sink | >300, >300^a^ | >300, >300 | 251, 270 | 48, 60 | 0, 0 | 11436 |
| AC07B | Air outlet | 40, 42 | 0, 0 | 0, 0 | 0, 0 | 0, 0 | 16 |
|  | Filter net | 39, 43 | 0, 0 | 0, 0 | 0, 0 | 0, 0 | 16 |
|  | Cooling fin | 37, 40 | 0, 0 | 0, 0 | 0, 0 | 0, 0 | 15 |
|  | Water sink | 42, 44 | 0, 0 | 0, 0 | 0, 0 | 0, 0 | 17 |
| AC08A | Air outlet | >300, >300^a^ | >300, >300 | 104,96 | <30, <30 | 0, 0 | 4000 |
|  | Filter net | >300, >300^a^ | >300, >300 | 168, 150 | <30, <30 | 0, 0 | 6360 |
|  | Cooling fin | >300, >300^a^ | >300, >300 | 133, 142 | <30, <30 | 0, 0 | 5500 |
|  | Water sink | >300, >300^a^ | >300, >300 | 270, 285 | 60, 53 | 0, 0 | 12145 |
| AC08B | Air outlet | 35, 40 | 0, 0 | 0, 0 | 0, 0 | 0, 0 | 15 |
|  | Filter net | 32, 36 | 0, 0 | 0, 0 | 0, 0 | 0, 0 | 14 |
|  | Cooling fin | 41, 47 | 0, 0 | 0, 0 | 0, 0 | 0, 0 | 18 |
|  | Water sink | 56, 52 | 0, 0 | 0, 0 | 0, 0 | 0, 0 | 22 |
| AC09A | Air outlet | >300, >300^a^ | >300, >300 | 130,135 | <30, <30 | 0, 0 | 5300 |
|  | Filter net | >300, >300^a^ | >300, >300 | 257, 264 | 52, 49 | 0, 0 | 11309 |
|  | Cooling fin | >300, >300^a^ | >300, >300 | 156, 173 | <30, <30 | 0, 0 | 6580 |
|  | Water sink | >300, >300^a^ | >300, >300 | 290, 277 | 63, 61 | 0, 0 | 12564 |
| AC09B | Air outlet | 54, 45 | 0, 0 | 0, 0 | 0, 0 | 0, 0 | 20 |
|  | Filter net | 46, 41 | 0, 0 | 0, 0 | 0, 0 | 0, 0 | 17 |
|  | Cooling fin | 36, 42 | 0, 0 | 0, 0 | 0, 0 | 0, 0 | 16 |
|  | Water sink | 56, 62 | 0, 0 | 0, 0 | 0, 0 | 0, 0 | 24 |
| AC10A | Air outlet | >300, >300^a^ | >300, >300 | 132, 129 | <30, <30 | 0, 0 | 5220 |
|  | Filter net | >300, >300^a^ | >300, >300 | 245, 266 | 43, 40 | 0, 0 | 10800 |
|  | Cooling fin | >300, >300^a^ | >300, >300 | 174, 168 | <30, <30 | 0, 0 | 6840 |
|  | Water sink | >300, >300^a^ | >300, >300 | 293, 290 | 85, 75 | 0, 0 | 13509 |
| AC10B | Air outlet | 38, 47 | 0, 0 | 0, 0 | 0, 0 | 0, 0 | 17 |
|  | Filter net | 42, 45 | 0, 0 | 0, 0 | 0, 0 | 0, 0 | 17 |
|  | Cooling fin | 36, 35 | 0, 0 | 0, 0 | 0, 0 | 0, 0 | 14 |
|  | Water sink | 46, 44 | 0, 0 | 0, 0 | 0, 0 | 0, 0 | 18 |

^a^ The counts of colony in the two parallel plates.
