# Supplementary material for: Bacterial contamination in the different parts of household air conditioners: a comprehensive evaluation from Chengdu, Southwest China
Source: Front Public Health. 2024 Aug 14;12:1429626. doi: 10.3389/fpubh.2024.1429626 (PMC11350112; doi:10.3389/fpubh.2024.1429626)
Supplement: Supplementary file 2 [file Table_2.docx]

S2. The data of total fungi count for all samples.

| Sample ID | Sampling part | Dilutability | | | | | Colony forming unit  (CFU/cm^2^, round to single digits^)^ | |
| --- | --- | --- | --- | --- | --- | --- | --- | --- |
|  |  | 10^-1^ | 10^-2^ | 10^-3^ | 10^-4^ |  | |  |
| AC01A | Air outlet | >50, >50^a^ | >50, >50 | 16, 10 | 0, 0 | 520 | |  |
|  | Filter net | >50, >50 | >50, >50 | 30, 32 | 0, 0 | 1240 | |  |
|  | Cooling fin | >50, >50 | >50, >50 | 22, 25 | 0, 0 | 940 | |  |
|  | Water sink | >50, >50 | >50, >50 | 36, 39 | 0, 0 | 1500 | |  |
| AC01B | Air outlet | 15, 21 | 0, 0 | 0, 0 | 0, 0 | 7 | |  |
|  | Filter net | 24, 25 | 0, 0 | 0, 0 | 0, 0 | 10 | |  |
|  | Cooling fin | 20, 22 | 0, 0 | 0, 0 | 0, 0 | 17 | |  |
|  | Water sink | 30, 27 | 0, 0 | 0, 0 | 0, 0 | 11 | |  |
| AC02A | Air outlet | >50, >50 | >50, >50 | 25, 21 | 0, 0 | 920 | |  |
|  | Filter net | >50, >50 | >50, >50 | 40, 42 | 0, 0 | 1640 | |  |
|  | Cooling fin | >50, >50 | >50, >50 | 32, 35 | 0, 0 | 1340 | |  |
|  | Water sink | >50, >50 | >50, >50 | 44, 46 | 0, 0 | 1800 | |  |
| AC02B | Air outlet | 23, 31 | 0, 0 | 0, 0 | 0, 0 | 10 | |  |
|  | Filter net | 27, 26 | 0, 0 | 0, 0 | 0, 0 | 11 | |  |
|  | Cooling fin | 18, 20 | 0, 0 | 0, 0 | 0, 0 | 8 | |  |
|  | Water sink | 30, 33 | 0, 0 | 0, 0 | 0, 0 | 13 | |  |
| AC03A | Air outlet | >50, >50 | >50, >50 | 12, 16 | 0, 0 | 560 | |  |
|  | Filter net | >50, >50 | >50, >50 | 25, 29 | 0, 0 | 1080 | |  |
|  | Cooling fin | >50, >50 | >50, >50 | 15, 14 | 0, 0 | 580 | |  |
|  | Water sink | >50, >50 | >50, >50 | 31, 24 | 0, 0 | 1100 | |  |
| AC03B | Air outlet | 22, 23 | 0, 0 | 0, 0 | 0, 0 | 9 | |  |
|  | Filter net | 25, 30 | 0, 0 | 0, 0 | 0, 0 | 11 | |  |
|  | Cooling fin | 10, 14 | 0, 0 | 0, 0 | 0, 0 | 5 | |  |
|  | Water sink | 24, 27 | 0, 0 | 0, 0 | 0, 0 | 10 | |  |
| AC04A | Air outlet | >50, >50 | >50, >50 | 23, 28 | 0, 0 | 1020 | |  |
|  | Filter net | >50, >50 | >50, >50 | 38, 44 | 0, 0 | 1640 | |  |
|  | Cooling fin | >50, >50 | >50, >50 | 26, 22 | 0, 0 | 960 | |  |
|  | Water sink | >50, >50 | >50, >50 | 40, 45 | 0, 0 | 1700 | |  |
| AC04B | Air outlet | 32, 23 | 0, 0 | 0, 0 | 0, 0 | 11 | |  |
|  | Filter net | 29, 33 | 0, 0 | 0, 0 | 0, 0 | 12 | |  |
|  | Cooling fin | 25, 28 | 0, 0 | 0, 0 | 0, 0 | 11 | |  |
|  | Water sink | 31, 36 | 0, 0 | 0, 0 | 0, 0 | 13 | |  |
| AC05A | Air outlet | >50, >50 | >50, >50 | 33, 30 | 0, 0 | 1260 | |  |
|  | Filter net | >50, >50 | >50, >50 | 40, 44 | 0, 0 | 1680 | |  |
|  | Cooling fin | >50, >50 | >50, >50 | 31, 36 | 0, 0 | 1340 | |  |
|  | Water sink | >50, >50 | >50, >50 | 44, 48 | 0, 0 | 1840 | |  |
| AC05B | Air outlet | 21, 26 | 0, 0 | 0, 0 | 0, 0 | 9 | |  |
|  | Filter net | 23, 25 | 0, 0 | 0, 0 | 0, 0 | 10 | |  |
|  | Cooling fin | 40, 32 | 0, 0 | 0, 0 | 0, 0 | 14 | |  |
|  | Water sink | 26, 22 | 0, 0 | 0, 0 | 0, 0 | 10 | |  |

Table continued.

| Sample ID | Sampling part | Dilutability | | | | | Colony forming unit  (CFU/cm^2^, round to single digits^)^ | |
| --- | --- | --- | --- | --- | --- | --- | --- | --- |
|  |  | 10^-1^ | 10^-2^ | 10^-3^ | 10^-4^ |  | |  |
| AC06A | Air outlet | >50, >50 | >50, >50 | 9, 15 | 0, 0 | 480 | |  |
|  | Filter net | >50, >50 | >50, >50 | 27, 30 | 0, 0 | 1140 | |  |
|  | Cooling fin | >50, >50 | >50, >50 | 25, 27 | 0, 0 | 1040 | |  |
|  | Water sink | >50, >50 | >50, >50 | 44, 40 | 0, 0 | 1680 | |  |
| AC06B | Air outlet | 32, 34 | 0, 0 | 0, 0 | 0, 0 | 13 | |  |
|  | Filter net | 30, 32 | 0, 0 | 0, 0 | 0, 0 | 12 | |  |
|  | Cooling fin | 25, 29 | 0, 0 | 0, 0 | 0, 0 | 11 | |  |
|  | Water sink | 25, 31 | 0, 0 | 0, 0 | 0, 0 | 11 | |  |
| AC07A | Air outlet | >50, >50 | >50, >50 | 14, 24 | 0, 0 | 760 | |  |
|  | Filter net | >50, >50 | >50, >50 | 31,30 | 0, 0 | 1220 | |  |
|  | Cooling fin | >50, >50 | >50, >50 | 25, 21 | 0, 0 | 920 | |  |
|  | Water sink | >50, >50 | >50, >50 | 42, 33 | 0, 0 | 1500 | |  |
| AC07B | Air outlet | 15, 12 | 0, 0 | 0, 0 | 0, 0 | 5 | |  |
|  | Filter net | 16, 20 | 0, 0 | 0, 0 | 0, 0 | 7 | |  |
|  | Cooling fin | 21, 24 | 0, 0 | 0, 0 | 0, 0 | 9 | |  |
|  | Water sink | 16, 28 | 0, 0 | 0, 0 | 0, 0 | 9 | |  |
| AC08A | Air outlet | >50, >50 | >50, >50 | 14, 8 | 0, 0 | 440 | |  |
|  | Filter net | >50, >50 | >50, >50 | 33, 40 | 0, 0 | 1460 | |  |
|  | Cooling fin | >50, >50 | >50, >50 | 23, 29 | 0, 0 | 1040 | |  |
|  | Water sink | >50, >50 | >50, >50 | 40, 45 | 0, 0 | 1700 | |  |
| AC08B | Air outlet | 7, 10 | 0, 0 | 0, 0 | 0, 0 | 3 | |  |
|  | Filter net | 26, 24 | 0, 0 | 0, 0 | 0, 0 | 10 | |  |
|  | Cooling fin | 26, 25 | 0, 0 | 0, 0 | 0, 0 | 10 | |  |
|  | Water sink | 30, 25 | 0, 0 | 0, 0 | 0, 0 | 11 | |  |
| AC09A | Air outlet | >50, >50 | >50, >50 | 20, 33 | 0, 0 | 1060 | |  |
|  | Filter net | >50, >50 | >50, >50 | 45, 40 | 0, 0 | 1700 | |  |
|  | Cooling fin | >50, >50 | >50, >50 | 32, 27 | 0, 0 | 1180 | |  |
|  | Water sink | >50, >50 | >50, >50 | 48, 44 | 0, 0 | 1840 | |  |
| AC09B | Air outlet | 11, 16 | 0, 0 | 0, 0 | 0, 0 | 5 | |  |
|  | Filter net | 17, 14 | 0, 0 | 0, 0 | 0, 0 | 6 | |  |
|  | Cooling fin | 21, 23 | 0, 0 | 0, 0 | 0, 0 | 9 | |  |
|  | Water sink | 30, 31 | 0, 0 | 0, 0 | 0, 0 | 12 | |  |
| AC10A | Air outlet | >50, >50 | >50, >50 | 24, 34 | 0, 0 | 1160 | |  |
|  | Filter net | >50, >50 | >50, >50 | 46, 45 | 0, 0 | 1820 | |  |
|  | Cooling fin | >50, >50 | >50, >50 | 35, 38 | 0, 0 | 1460 | |  |
|  | Water sink | >50, >50 | >50, >50 | 45, 47 | 0, 0 | 1840 | |  |
| AC10B | Air outlet | 11, 13 | 0, 0 | 0, 0 | 0, 0 | 5 | |  |
|  | Filter net | 25, 29 | 0, 0 | 0, 0 | 0, 0 | 11 | |  |
|  | Cooling fin | 30, 25 | 0, 0 | 0, 0 | 0, 0 | 11 | |  |
|  | Water sink | 40, 33 | 0, 0 | 0, 0 | 0, 0 | 15 | |  |

^a^ The counts of colony in the two parallel plates.
