# Supplementary material for: Bacterial contamination in the different parts of household air conditioners: a comprehensive evaluation from Chengdu, Southwest China
Source: Front Public Health. 2024 Aug 14;12:1429626. doi: 10.3389/fpubh.2024.1429626 (PMC11350112; doi:10.3389/fpubh.2024.1429626)
Supplement: Supplementary file 3 [file Table_3.docx]

S3. The sample ID of 16S ribosomal RNA (rRNA) gene sequencing

|  | Household | | | | | | | | | |
| --- | --- | --- | --- | --- | --- | --- | --- | --- | --- | --- |
| Sampling parts | 1 | 2 | 3 | 4 | 5 | 6 | 7 | 8 | 9 | 10 |
| Air outlet | AC01A1 | AC02A1 | AC03A1 | AC04A1 | AC05A1 | AC06A1 | AC07A1 | AC08A1 | AC09A1 | AC10A1 |
| Filter net | AC01A2 | AC02A2 | AC03A2 | AC04A2 | AC05A2 | AC06A2 | AC07A2 | AC08A2 | AC09A2 | AC10A2 |
| Cooling fin | AC01A3 | AC02A3 | AC03A3 | AC04A3 | AC05A3 | AC06A3 | AC07A3 | AC08A3 | AC09A3 | AC10A3 |
| Water sink | AC01A4 | AC02A4 | AC03A4 | AC04A4 | AC05A4 | AC06A4 | AC07A4 | AC08A4 | AC09A4 | AC10A4 |
